# Harnessing repeated measurements of predictor variables for clinical risk prediction: a review of existing methods

**DOI:** 10.1186/s41512-020-00078-z

**Published:** 2020-07-09

**Authors:** Lucy M. Bull, Mark Lunt, Glen P. Martin, Kimme Hyrich, Jamie C. Sergeant

**Affiliations:** 1 Centre for Epidemiology Versus Arthritis, Centre for Musculoskeletal Research, Manchester Academic Health Science Centre, University of Manchester, Manchester, UK; 2 Centre for Biostatistics, Manchester Academic Health Science Centre, University of Manchester, Manchester, UK; 3 Division of Informatics, Imaging and Data Science, Faculty of Biology, Medicine and Health, University of Manchester, Manchester Academic Health Science Centre, Manchester, UK; 4 National Institute for Health Research Manchester Biomedical Research Centre, Manchester University NHS Foundation Trust, Manchester Academic Health Science Centre, Manchester, UK

**Keywords:** Longitudinal data, Clinical risk prediction, Dynamic prediction, Prediction models, Survival analysis, Repeated observations, Electronic health records, Personalised medicine, Time-dependent covariates, Joint models

## Abstract

**Background:**

Clinical prediction models (CPMs) predict the risk of health outcomes for individual patients. The majority of existing CPMs only harness cross-sectional patient information. Incorporating repeated measurements, such as those stored in electronic health records, into CPMs may provide an opportunity to enhance their performance. However, the number and complexity of methodological approaches available could make it difficult for researchers to explore this opportunity. Our objective was to review the literature and summarise existing approaches for harnessing repeated measurements of predictor variables in CPMs, primarily to make this field more accessible for applied researchers.

**Methods:**

MEDLINE, Embase and Web of Science were searched for articles reporting the development of a multivariable CPM for individual-level prediction of future binary or time-to-event outcomes and modelling repeated measurements of at least one predictor. Information was extracted on the following: the methodology used, its specific aim, reported advantages and limitations, and software available to apply the method.

**Results:**

The search revealed 217 relevant articles. Seven methodological frameworks were identified: time-dependent covariate modelling, generalised estimating equations, landmark analysis, two-stage modelling, joint-modelling, trajectory classification and machine learning. Each of these frameworks satisfies at least one of three aims: to better represent the predictor-outcome relationship over time, to infer a covariate value at a pre-specified time and to account for the effect of covariate change.

**Conclusions:**

The applicability of identified methods depends on the motivation for including longitudinal information and the method’s compatibility with the clinical context and available patient data, for both model development and risk estimation in practice.

## Background

Clinical prediction models (CPMs) aim to predict the risk of health outcomes such as disease onset, disease progression or likely outcomes of treatment [1]. Such predictions are based on available information about an individual at the time of prediction and can be used to inform patient care. This could be by offering preventative interventions to those predicted to be at high risk of an adverse outcome or relaxing the monitoring of those predicted to be at low risk. A clinical example of the former is the QRISK prediction tool currently used in primary care to estimate a patient’s 10-year risk of cardiovascular disease (CVD) [2]. UK health guidelines advise that anyone with an estimated CVD risk of 10% or higher (from the QRISK model) should be prescribed a statin to reduce their risk of CVD onset [2, 3].

The majority of current CPMs use patient information from only a single time point to make predictions and fail to take advantage of longitudinal medical data, such as that available in electronic health records (EHRs). It has been hypothesized that repeated observations provide a predictive advantage over cross-sectional information as they capture change in individual patients over time and are less sensitive to measurement error [4, 5]. Furthermore, recent empirical reviews comparing longitudinal CPMs to the traditional cross-sectional ones provide some evidence that the overall predictive accuracy can be improved by incorporating the longitudinal patient information [5–9].

While an increasing number of CPMs are being developed using EHR data, a systematic review showed that less than 9% of identified CPMs exploited the time-varying nature of their predictor variables [6]. Therefore, although methods for longitudinal data analysis are well established, they appear to be under-utilised in the development of CPMs.

To the authors’ knowledge, a broad review of available methods adopted for harnessing longitudinal data in binary or time-to-event CPMs has not yet been performed. Binary and time-to-event outcomes are of primary interest here as they are the most commonly reported amongst the prediction-modelling literature [6, 7]. Previous reviews have been restricted to simpler methods [8], methods most compatible to a particular clinical application [5, 9–11], or they have been restricted to the two commonly considered methods in the field of CPMs (i.e. joint models and landmark analysis, see Results for method description) [12–14]. The availability of a broad review could help the development of longitudinal CPMs and their potential use in practice.

Our primary objective was to review the literature and provide applied researchers with a comprehensive summary of existing approaches used for harnessing repeated measurements of predictors in CPMs. To address this objective, we sought to group identified methods based on their similarity and how they use repeated observations to enhance prediction, as well as outline their reported advantages and limitations. Our secondary objectives were to provide guidance on how to choose an appropriate method and to highlight opportunities for further methodological research.

## Methods

### Definitions and terminology

Within this review, longitudinal information is defined as repeated measurements through time of predictor variables. Predictor variables here are defined as measurable factors that are potentially predictive of health outcomes of interest. The terms `predictors’ and `covariates’ will also be used interchangeably for `predictor variables’. Note that the analytical methods discussed in this review are sometimes categorised under the term `dynamic prediction’. However, dynamic prediction can cover a broader range of aims and methods than those of interest here. In particular, the methods covered in this review are distinct to those for addressing calibration drift [15] or modelling disease state transitions [16].

### Search strategy

The search strategy in Table 1 was designed to find peer-reviewed journal articles that described the development of a CPM for individual-level prediction of a binary or time-to-event outcome, and accounted for repeated measurements over time of at least one predictor variable during model development.

**Table 1:** Search strategy in Ovid format, as entered into MEDLINE and Embase

1	((repeat* adj1 measure*) OR ``repeatedly-measured" OR (repeat* adj1 observ*) OR ``repeatedly-observed").ti,ab
2	``time-series" OR ``time-series" OR (``longitudinal" adj2 ``data")).ti,ab
3	``longitudinal" adj3 (``survival" OR ``binary")) OR ((``longitudinal" OR ``repeat" OR ``discrete") adj2 (``time-to-event" OR (event* adj2 time*)))).ti,ab
4	((time-depend* OR ``time-varying" OR ``longitudinal") adj1 (coefficient* OR variable* OR covariate* OR marker* OR factor* OR observ* OR measure* OR biomarker* OR model* OR predictor*)).ti,ab
5	((predict* adj1 (accurac* OR ``power" OR individual* OR ``future" OR ``time-to-event" OR (event* adj2 time*) OR ``binary")) OR ``predictive ability" OR ``predictive performance").ti,ab
6	(((``predictive" OR ``prediction" OR ``prognostic") adj1 (tool* OR scor* OR ``algorithm" OR model* OR rule*))).ti,ab
7	``predict" or ``predicts" or ``prediction" or ``predicting") adj2 (risk* OR ``outcome" OR ``incidence" OR ``time" OR development* OR event* OR ``disease" OR recurrence* OR ``progression" OR ``severity" OR ``achievement" OR ``status" OR ``application")).ti,ab
8	((``predict" or ``predicts" or ``prediction" or ``predicting") adj3 (``mortality" OR ``survival")).ti,ab
9	(``dynamic prediction" OR ``dynamic predictions" OR ``dynamic prognostic" OR ``dynamic clinical prediction").ti,ab
10	1 OR 2 OR 3 OR 4
11	5 OR 6 OR 7 OR 8
12	10 AND 11
13	12 OR 9

The search terms (Table 1) were entered into MEDLINE (via Ovid), Embase (via Ovid) and Web of Science. The search was restricted to peer-reviewed journal articles in English. Further details about any refinements specific to each database have been reported in Table 2. Duplicates were removed using automatic deduplication on both EndNote X8 and Mendeley Desktop.

**Table 2:** Search limits specific to MEDLINE, Embase and Web of Science

Search limits			
Database	Ovid MEDLINE(R) and Epub Ahead of Print, In-process and Other Non-indexed Citations, Daily and Versions(R)	Ovid Embase	Web of Science
Search	Title and Abstract (.ti,ab)	Title and Abstract (.ti,ab)	Title, abstract, and keywords (TS=())
Dates	1946 to November 30, 2018	1974 to 2018 December 03	1900–2018
Publication status	No limit	Article-in-press, Embase status, In-Process status	No limit
Document type	Journal Article	Article, Article in press	Article
Language	English	English	English
Categories	No limit	No limit	Categories covering computer science, biology, healthcare, pharmacy, mathematics, statistics, biomedical engineering, psychology and probability.
Citation index	No limit	No limit	Social science, Science, Emerging sources.

### Inclusion and exclusion criteria

A two-stage screening process was performed prior to full-text assessment for eligibility. Different sets of inclusion criteria were used to screen titles and abstracts, set A and set B respectively. Set B was also used for full-text assessment. Both sets of inclusion criteria are clearly stated, alongside the rule of inclusion, in Table 3. For an article to be taken through a stage of the screening process (or the full-text assessment), it must have satisfied the `rule of inclusion’ (e.g. article titles that did not satisfy either criterion 1 or 2 alongside criterion 3 in set A were excluded from the review). If it was unclear whether an article satisfied the inclusion criteria in the relevant set, it was automatically brought forward to the next stage (i.e. to abstract screening or full-text assessment).

**Table 3:** Inclusion criteria used for the title, abstract and full-text screening

	Inclusion criteria set A (for titles)	Inclusion criteria set B (for abstracts and full-texts)
**1**	Development of a CPM.	Development of a multivariable CPM, which predicts a binary or time-to-event outcome.
**2**	Modelling techniques for longitudinal and survival/binary data.	CPM accounts for repeated measurements over time of at least one predictor variable.
**3**	Clinical application described or article published in a medical or biometric journal.	CPM has been developed for a binary or time-to-event outcome for an individual*.*
Inclusion rule	(1 OR 2) AND 3	1 AND 2 AND 3

### Information extraction

The following information was extracted from relevant journal articles: the method for modelling longitudinal predictor variables, the aim of the method, the computer software used (if stated), the number and type of variables modelled longitudinally within the CPM, the clinical application and publication year. Publication years were extracted to provide a graphical overview of method usage over time. For any methods identified during the search, reported advantages, challenges and opportunities for future work regarding their application in CPMs were also extracted.

## Results

### Database search

**Fig. 1:**
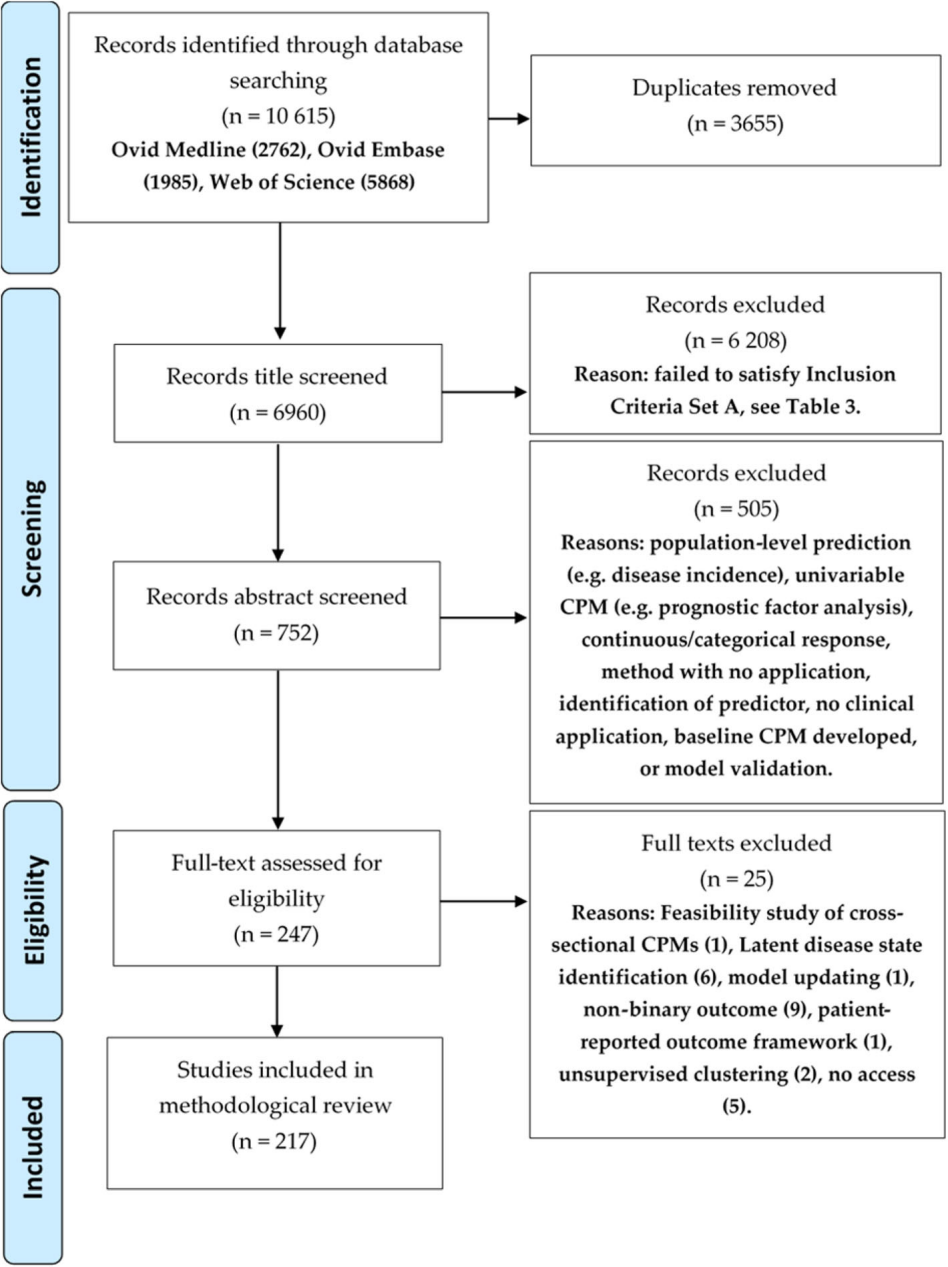
PRISMA flow diagram to illustrate the screening process

The database search produced 10 615 results, which included 6960 unique peer-reviewed journal articles after the removal of duplicates, book chapters and conference proceedings. Following title screening, 752 articles remained in the review. The abstract screening and full-text assessment for eligibility left 247 and 217 articles respectively. The full screening process and reasons for exclusion have been described in Fig. 1. Additional file 1 lists all the articles included in the review.

### Methodological review

**Fig. 2:**
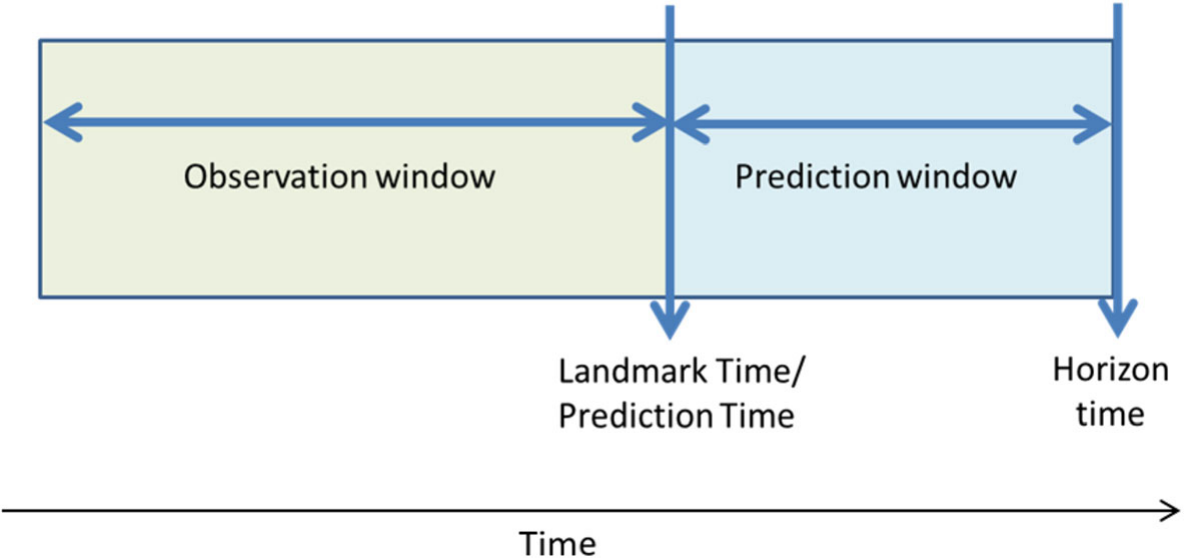
Temporal terminology for harnessing repeated measurements of predictors in clinical prediction models

The following terminology was identified within the review, which we here define to facilitate the understanding and comparison of methods described below: landmark time, prediction time, horizon time, observation window and prediction window (see Fig. 2). Landmark time is equivalent to prediction time, which is the time that an individual’s prognosis is being assessed. Horizon time is the end of the period that the prediction applies to. As an example, the QRISK models are developed to predict 10-year risk of cardiovascular disease, the horizon time is thus landmark time + 10 years [2]. Observation window refers to the period of time where a patient’s covariate history can be observed for the purpose of inclusion into the CPM, which is always prior and up until the landmark/prediction time. Finally, the prediction window is the time period between the landmark time and horizon time.

From the included studies, three distinct methodological aims for harnessing repeatedly measured predictors in the development of CPMs were identified. All of the discovered methods satisfied one or more of these three methodological aims: (A1) to better represent the predictor-outcome relationship, (A2) to infer or predict a covariate value at a pre-specified time or (A3) to account for the effects of how a predictor changes over time. It is important to highlight that the content of this methodological review only covers methods reported in the identified literature via the database search, and that other valid approaches may exist but have not yet been applied in this field of clinical risk prediction.

Methods satisfying A1 tend to utilise repeated observations to represent a time-constant relationship, or better represent a time-varying relationship, between a predictor and the event of interest. Consequently, these methods often also allow for updated predictions through time. A2 is often pursued to either account for measurement error or random noise, or to impute missing data when measurements are irregularly-spaced. Methods for A3 are adopted when it is the behaviour of the covariate that is considered predictive of the event of interest.

**Fig. 3:**
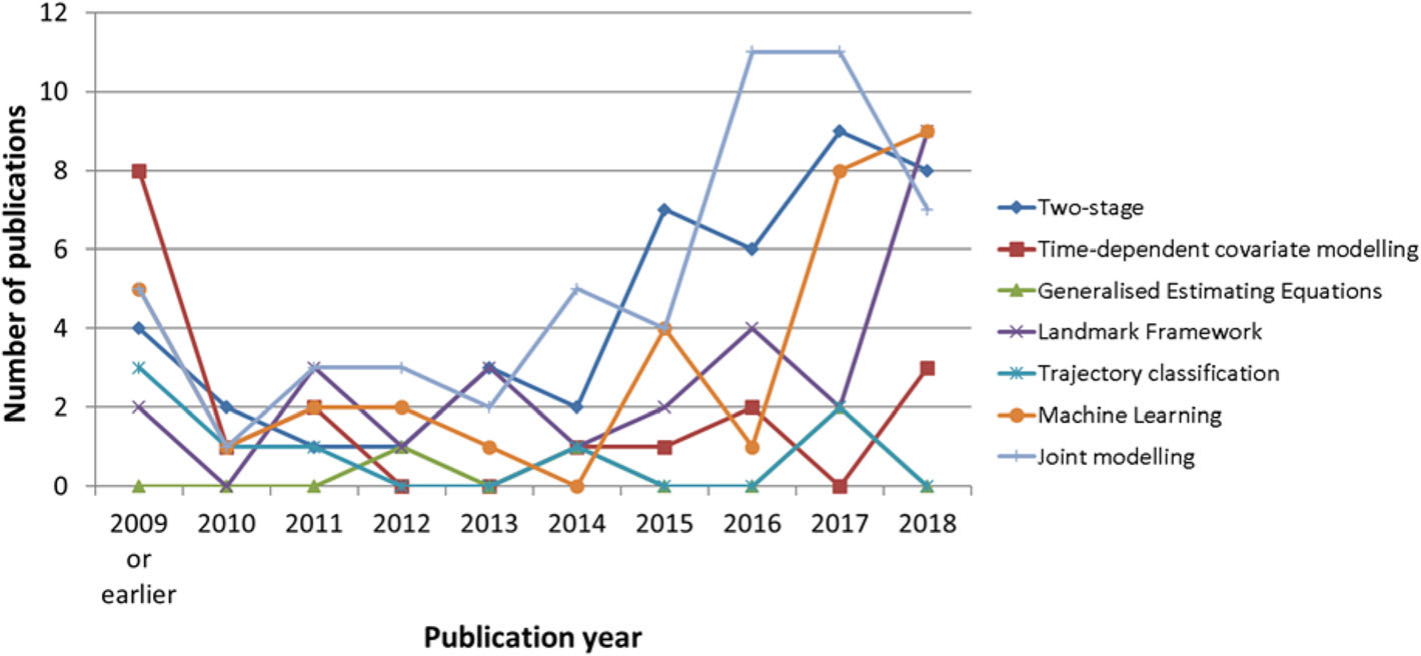
Number of publications per year for each framework (*n* = 182), excluding methods in comparative reviews

In addition to identifying the three aims, the available methods were categorised into seven distinct frameworks: time-dependent covariate modelling (TDCM), generalised estimating equations (GEE), landmark analysis (LA), two-stage modelling (TSM), joint-modelling (JM), trajectory classification (TC) and machine learning (ML). All identified methods require subject-level longitudinal information on a study population for CPM development. The TSM, JM and TC frameworks (as well as some ML algorithms) can also harness a subject’s repeated measurements at the time of prediction. Meanwhile, as stand-alone frameworks, the TDCM, GEE and LA frameworks only require a subject’s most recent observations (i.e. a maximum of one measurement for each predictor) at the time of prediction. Figure 3 provides an overview of framework adoption over the past decade, showing that the JM, TSM, LA and ML frameworks appear to be the most popular. GEE and TC frameworks are the least adopted frameworks.

Some of the above frameworks have been extended to harness `functional data’, defined by Li and Luo [17] as data that ‘provide information about curves, surfaces, or anything else varying over a continuum’. For our review, this includes functional data on both a one-dimensional time domain such as heart rate monitor data or electroencephalogram data, and on higher dimensional domains such as magnetic resonance imaging or positron emission tomography. Any extensions of methods for functional data will be specified below. Multivariate longitudinal methods for prediction tailored to functional data generated in a critical care setting were also outlined by Plate et al. in 2019 [11]. The remainder of this subsection provides a detailed description of each identified methodological framework in turn. The description includes how they use longitudinal information, their reported advantages and limitations, and their extensions. An overview of each framework, their corresponding available software and example clinical applications is reported in Table 4**.**

**Table 4:** Methodological frameworks available to enhance clinical prediction models using longitudinal information

Framework	Aim	Advantages	Limitations	Software	Extensions/variations	Examples
1. Time-dependent covariate modelling (TDCM)	A1	Allows for updated predictions over time, simple to apply in available software.	Assumes no measurement error, cannot predict the future, correlations^a^ ignored, measurements assumed constant between time-points, requires complete predictors at event times.	Widely available (e.g. R, Stata, SAS).	Time-varying effects [25], time-since-measurement as a predictor [27], aggregated covariate [26]. Quantile residual life regression [109].	Applied to assess the prognosis of patients with hepatocellular carcinoma, allowing for prediction at any stage of disease using their most recent information [110].
2. Generalised estimating equations (GEE)	A1	Allows for updated predictions over time, accounts for correlation^a^, can adjust for patient clustering.	Ignores underlying trajectory, does not account for changes in at-risk population, and ignores time-dependency.	Widely available (e.g. R, stata, SAS). *geepack* package on R.		Employed to identify patients at high risk of adverse events after cancer therapy [28–30]. To account for repeated pre-therapy measurements and outcomes per individual though repeated treatment cycles.
3. Landmark analysis (LA)	A1	Avoids misspecification of underlying trajectory, only uses patient information prior to landmark time.	Ignores underlying covariate trajectory, often correlations^a^ ignored, requires complete follow-up, and LOCF approach induces bias.	*dynpred* and *coxph* functions in R.	Competing risks [34, 41], recurrent events [36], combined with TSM [34, 38, 40], pseudo-observations [35, 41], cure fraction models [42].	Employed to predict relapse/death for those in leukaemia remission after transplant [34]. Landmark times 1, 6 and 12 months after bone marrow transplant [34]. Accounted for complications experienced by patients during follow-up.
4. Two-stage modelling (TSM)	A2 or A3	Simple to apply, flexible, can account for correlations^a^, can handle irregularly spaced measurements.	Ignores model-specification error in the first-stage, first model cannot account for drop-out bias.	*refund* in MFPCA R package (FPC), *merlin* package on R (ME models).	Extends to TDCM [111], and LA [112], calibration error included in stage II [52, 60].	In conjunction with LA, TSM used to predict adverse events following endovascular abdominal aortic aneurysm repair [44]. ME models for aneurysm sac diameter change over time, with Cox model [44].
5. Joint-modelling (JM)	A1 and (A2 OR A3)	Address limitations of TSM framework, allows updated predictions over time, flexible.	Complex to implement, strong parametric assumptions, computationally intensive.	*JMbayes or JM* R packages. *lcmm* R package for JLCMs, *frailtypack R* package for JFMs.	Time-varying effects [13], Bayesian moving average [53, 107], various functions of random effects [13, 53, 73–75, 113], third JM to handle missing data and cure fraction models [66, 114–116].	Shared random effects JM employed for real-time predictions of prostate cancer recurrence [74]. A ME sub-model for log PSA over time, and a Cox sub-model used for the time-to-event outcome. Estimated using MCMC.
6. Trajectory classification (TC)	A1, A2 and A3	Accounts for correlation^a^, irregularly-spaced measurements, informative processes, updated predictions, underlying trajectory.	Complex and computationally intensive for multivariate applications, parametric assumptions required for covariate trajectory.	*merlin* package on R (ME models), *Rstan* package (Gaussian processes).	Multivariate modelling using Gaussian processes [117]. Multivariate modelling and informative processes [76]	Employed to classify repeated measurements of hormone levels in early pregnancy to predict pregnancy success in the context of in vitro fertilization [54]. A nonlinear ME model for hormone levels over time. The binary outcome (pregnancy) modelled as an interaction.
7. Machine learning (ML)	A1 and A3	Few assumptions, handles high-dimensional data, can identify optimal trajectory characteristics.	Often predicts binary outcome, ignores right-censoring, large datasets required to avoid overfitting, often ‘black box’ algorithms.	*random-Forest* R package, *Adaboost* or *gbm* R packages for Boosting, *LibSVM* on R for SVMs.	Recurrent Neural Networks (RNNs) [98–101], Multiple measurements and time series SVM [118, 119], ME models and conditional inference trees [81].	RNNs employed to predict heart failure based on EHR data [98]. RNN identified patterns in previous and current diagnoses and quantified similarities with historic patients diagnosed with heart failure [98].

^a^Correlations between and within individuals

*Abbreviations*: *LOCF* last observation carried forward, *ME* mixed effect, *SVM* support vector machine, *MCMC* Markov chain Monte Carlo, *EHR* electronic health record, *JLCM* joint latent class model

#### Time-dependent covariate modelling

The most prominent approach before 2009 was to include time-dependent covariates within a survival model [5, 18]. We refer to this technique as the TDCM framework*,* as it can be applied to various adaptations of Cox regression models. The TDCM framework allows for instantaneous risk estimates to be produced at any time within the observation window and prediction window conditional on survival up until that time, whilst harnessing an individual’s most recent observations [5, 19–21]. Conceptually, this approach compares the most recent covariate values for those still at risk just before each event time for those who have and have not experienced an event at that specific time. From there, the hazard function is updated over time and a time-constant effect between each covariate and the event of interest is estimated [5]. Therefore, TDCM falls under the first methodological aim (A1) and, as the timing of each event is required, can only handle time-to-event outcomes.

TDCM provides an advantage over baseline CPMs by enabling risk estimates to be updated during follow-up for new individuals, using their most recent covariate values [22]. Applying baseline CPMs to patient data collected during follow-up would lead to under-estimated risk predictions and over-estimated survival predictions [22]. However, TDCM has been heavily criticised throughout the literature for the following reasons. First, covariate values are assumed to be measured without error [5, 21]. Second, repeated covariate values over time are assumed to remain constant between data collection points [5, 21]. Third, correlations between and within subject measurements are not taken into account [21]. Finally, and most importantly, a time-dependent survival model is unable to predict into the future beyond the first change in the covariates [21, 23].

To elaborate on this final limitation, the challenge lies with the requirement of patient covariate values at the horizon time, as these are unknown for new individuals in practice. The simplest, and most common approach, to overcoming this final limitation is to use last observation carried forward (LOCF) from landmark time to horizon time [5]. This variation of TDCM has been employed, for example, to assess the prognosis of individuals with hepatocellular carcinoma at any stage of their disease using their most recent clinical information (Table 4) (103). The magnitude of the error introduced by the LOCF aspect of prediction for a new individual is usually dependent on the prediction window size and the stability of predictor variables over time, with TDCM being argued as a valid approach for short-term prediction windows [24].

Extensions of TDCM can account for time-dependent effects of predictors [25, 26], and aim to minimise the error caused by the LOCF approach by including time since measurement as a predictor [27], or including aggregated summaries of covariates [26].

#### Generalised estimating equations

Similarly to the TDCM framework, the primary methodological aim of generalised estimating equations (GEE) is to utilise repeated observations from the same individual to better represent the association between the predictor variables and the event of interest. However, unlike the TDCM framework, GEE models account for within and between individual correlation, can directly harness repeated events per individual [28, 29], and can model either binary or survival outcomes. In general, GEE models are most suitable when the model development data violates the independence assumption and the model developer’s primary interest is in the most accurate estimation of the predictor-outcome relationship.

More specifically for clinical risk prediction, GEEs have been employed to handle repetitions of cross-sectional patient information through time (both baseline and outcome information), which will here be referred to as `cycles’ of patient information [28–30]. As an example, a patient may experience several cycles of the same treatment (e.g. chemotherapy) to treat their condition (e.g. cancer); therefore, multiple pre-treatment measurements and multiple post-treatment adverse outcomes per patient could be utilised to develop a CPM for predicting adverse outcomes from chemotherapy [28–30].

Traditional logistic regression would not be able to utilise such patient information as it violates the independence assumption, that each observation (for an individual) is independent of other observations. An alternative model to handle cycles of patient information is a beta-geometric model that has been used to predict natural conception for women, after multiple cycles of a relevant procedure [31].

#### Landmark analysis

The landmark analysis (LA) framework derives separate cross-sectional CPMs for those still at risk at various landmark time points during follow-up. The flexibility in choice of model used to develop the CPM at each landmark time point allows for both binary and survival outcomes to be modelled under this framework. The methodological goal of this framework, which is similar to TDCM and based on conditional survival modelling [32], is to acknowledge that those who have survived for longer are more likely to have a better prognosis than those who have not [32]. Unlike TDCM, LA can use past or current information from new individuals to make predictions about their future [33]. The CPMs developed at each landmark time post-baseline can take into account the covariate history until the landmark time point, but a patient’s most recent observation is employed in time-fixed CPMs [28]. For example, the LA framework has been adopted with binary time-dependent covariates to predict relapse or death for those in remission from leukaemia after a bone marrow transplant [34], as shown in Table 4. The CPMs developed post-baseline acknowledged whether the patient had or hadn’t experienced complications since their surgery [34]. LA as a stand-alone framework utilises longitudinal information to account for change in an at-risk population when specifying the predictor-outcome relationship, and therefore satisfies the first methodological aim (A1).

For CPM development, it is common to merge all risk-sets (i.e. data required to develop a CPM at each landmark time) into a stacked dataset and fit just one model to the available data, including landmark time as an independent variable. This is often referred to as the `super landmark model’ [34, 35]. Correlations between the within-subject observations can be accounted for using GEEs [35], and non-parametric time-varying coefficients can be modelled over landmark time points [36, 37].

The LA framework is a simple way to update risk predictions over time, without imposing too many assumptions on the available information, and it can handle a large number of time-dependent covariates [36–39]. Its simplicity may also lead it to being more robust to misuse in practice as it is straightforward to implement and interpret by the end-users [36, 39].

However, it appears that there is no general guidance on the choice of landmark times as they vary with each application. Examples include using quantiles of event times to capture the changes in the at-risk population [35] or using different follow-up appointment times in clinical practice [34]. Implementation can also be challenging for left-censored information, and routinely collected data with no defined baseline time-point [37]. CPMs developed in routinely collected data have used age as the landmark time to overcome this barrier [38]. Furthermore, the LA framework carries the same limitations as any conditional survival model, which is the requirement of a large dataset, complete with long-term follow-up covariate and event information for each of the landmark time points [32].

Mixed-effects or auto-regressive time series models can also be used to capture a subject’s covariate trajectory and predict the value of a covariate at each landmark time point [23, 34, 37, 38, 40]. Various survival models have also been applied to account for competing risks [41], recurrent events [36] and cure fraction models [42]. Thus, variations of the LA framework may fall under the second and third methodological aim (A2 and A3) if combined with the two-stage framework (discussed below).

#### Two-stage modelling

The two-stage modelling (TSM) framework considers the modelling processes for repeated measurements (longitudinal model) and outcome prediction (survival or binary-outcome model) separately. A parameter estimate from the longitudinal model is included as a fixed-time covariate in the survival (or binary-outcome) model. TSM as a stand-alone framework is often employed to satisfy the second or third methodological aim, depending on whether it is the behaviour of the predictor which is placed into the second model (A3), or the predicted value of a covariate at a pre-specified time (A2).

The key advantage of this approach is that it is computationally efficient, especially compared to joint modelling [21, 43]. However the two stages are performed separately and acknowledgment of any error in the estimation process for the longitudinal model is not carried forward into the outcome prediction model [5]. Therefore, any resulting predictions could appear too precise [5].

In the TSM framework, many different statistical models could be applied at each of the two stages in the CPM development process. The simplest and most common approaches applied for the first stage are to aggregate the repeated observations into a summary statistic or to fit a mixed-effect model (described below)*.* Other examples include functional principal components and time series; these methods are described in Additional file 2. Examples of the survival or binary outcome models include Cox proportional hazards [44], logistic regression [45, 46] and partly conditional models [43].

##### Aggregated data

In the aggregated data approach, all available covariate information up until prediction time is aggregated into a summary statistic. Examples include the use of the cumulative mean, rate of change, standard deviation or variance, coefficient of variation or the minimum/maximum value of available measurements for each individual [2, 5, 8, 47–49]. The most common statistic for models developed on EHR data was the extreme (min/max) value of a predictor within a pre-specified observation window [50].

This approach attempts to minimise the effect of measurement error on individual risk predictions by summarising over the longitudinal trajectory. The clear advantages of this approach are the simplicity, lack of computational demand, reduced sensitivity to noisy data and ability to handle multiple repeatedly-measured predictors [9]. However, the unbiased estimates of the mean, standard deviation and variance assume no underlying trend, bias, or variability change in the process, which is unrealistic for most clinical data [5].

##### Mixed-effect models

Mixed-effects (ME) models can also be referred to as random-effects, hierarchical or multi-level models. Their name derives from the idea that population-level information is used to support and enhance power for subject-level inference where individual measurements may be minimal. Population-level information is captured in fixed effects, and subject-level variations from the population are captured in random effects [9, 51]. These include linear mixed models [5, 51, 52] and generalised linear mixed models [9, 13, 40, 53].

ME models can be used to represent the longitudinal trajectory of a predictor variable over time, and may or may not include additional predictors for the longitudinal predictor outcome. The random effects from this model, which reflect individual-level rate of change or an inferred value of a predictor variable (at a pre-specified time), could be included into a cross-sectional CPM [46]. For example, a linear mixed model (LMM) has been employed to represent a patient’s aneurysm sac diameter change over time [44]. Using this LMM, each new patient’s aneurysm sac diameter and its rate of growth can be estimated at the landmark time (using their previous measurements). These values were then used as predictors in a Cox survival model to estimate their risk of an adverse event [44]. This clinical example also explores LA and TSM combined (Table 4) [44]**.**

Although ME models are extremely flexible, challenges arise with correctly specifying a parametric trend over time and how to represent the individual rate of change in the final CPM. Linear models, quadratic growth curves [8, 52], fractional polynomials [44] and cubic splines [9, 13, 53] can be used to model the trend over time. Most CPM developers have adopted trends from previous literature in their specific field, but an appropriate model could also be found using data–driven techniques like the multiple fractional polynomial algorithm [44]. ME models can be extended to have t-distributed residuals with continuous outcomes to better handle outlier observations, and within-person correlations for the repeated measurements [54, 55]. They can also be extended to account for sub-groups within a population using latent class methods [56].

#### Joint modelling

The joint-modelling (JM) framework addresses the limitations of the TSM framework by simultaneously estimating the longitudinal sub-model and the survival or binary outcome sub-model [13, 51, 52]. The term “joint model” more broadly refers to any number of statistical models estimated jointly, but here the literature focussed on jointly modelling a longitudinal model and a survival or binary outcome model. Similar to the TSM framework, a ME model was often employed for the covariate trajectory and a Cox proportional hazards model for a time-to-event outcome [57]. However, variations of the event prediction sub-model exist in the CPM literature such as binary event models [52, 58–61], parametric survival models [9], models for discrete-time data [9, 62, 63], models for competing risks [64], generalised linear models [58], and models for left-truncated data [65, 66]. Furthermore, the ME models could be for different types of data (e.g. functional data) [66, 67], modelling nonlinear functions [68], modelling nonparametric functions [69, 70] or linear quantile mixed models [71] depending on the clinical context.

Under all JM frameworks, the various sub-models typically involve shared random effects, or latent variables, whether they are continuous or discrete [5, 9]. For the purposes of clinical risk prediction, three different frameworks have been described: shared random effects (SRE) joint models, joint latent class models (JLCM) and joint frailty models for recurrent events (JFM). A clinical example of where a SRE joint model has been employed to predict prostate cancer recurrence has been highlighted in Table 4. Detailed descriptions of these sub-categories and their differences can be found in Additional file 2.

One challenge of using random effects in CPMs is estimating the risk of a future event for a new subject, as their random effects are unknown. To resolve this, random effects can be sampled from their posterior predictive distribution, which is based on the population-level distribution of random effects from the fitted joint model, the new subject’s covariate values until the time of prediction, and conditional on the subject still being at risk at the time of prediction [72, 73].

A more popular choice is to employ the Monte Carlo simulation approach as it takes into account the uncertainty around the survival or event probability estimate [57, 72, 73]. Monte Carlo simulation is, conceptually, a procedure that repeatedly samples parameter estimates and random effects based on their estimated posterior distributions from the fitted joint model [72, 73]. A new individual’s random effects can be simulated from their posterior predictive distribution, as stated above [72, 73]. Repeatedly sampling from the posterior distributions allows for an empirical distribution around the estimated survival or event probability [73]. Monte Carlo simulation has been employed independently of the model estimation process and is reported to be computationally efficient in contrast to the joint model specification [74, 75].

#### Trajectory classification

Mixed-effect (ME) models have also been employed to classify longitudinal trajectories for binary events or categories; these methods have been grouped under the trajectory classification framework for this review [52, 76, 77]. The methods can alternatively be referred to as “longitudinal linear discriminant analysis” or “pattern mixture models” depending on their estimation and classification process.

In the TC framework for the prediction of binary events, the binary outcome value of 0 or 1 is seen as a latent class variable in the mixed-effect model. That is, the outcome variable interacts with all the predictors within the mixed-effects model (both fixed and random), which specifies the longitudinal trajectories. For CPM development, events are observed and so the parameters can be estimated, which is like modelling the event and non-event subjects separately. In practice, when the outcome is unknown, separate distributions of the longitudinal predictor values can be estimated based on the *event* and *non-event* ME model parameters, as well as the new subject’s observed longitudinal values [78–80]. Both of these distributions can then be used to produce a discrimination score, which can later be used to classify the subject or to produce a posterior probability that the subject will experience the event [78–80].

A subject’s risk or discriminant score can also be re-estimated when new information becomes available. Therefore, this framework satisfies both the first and third methodological aim (A1 and A3). To extend this approach to predict time-to-event outcomes, covariate trajectories may be classified into categories that can then be used as a predictor within a survival model. This extension can be performed under the two-stage modelling or joint-modelling framework, the latter approach is referred to as the joint latent class model in Additional file 2. The TC framework has also been extended to incorporate additional models to account for repeated binary events over time, and for informative processes [76].

#### Machine learning

The definition of the term *machine learning* can often be ambiguous as it covers a broad range of data-driven algorithms in the fields of statistics and computer science. For the purpose of prediction, ML algorithms extend from regression-based models (such as logistic regression) to more complex mathematical modelling (such as neural networks). Although methods identified under the field of machine learning are not independent of regression-based techniques described elsewhere, what distinguishes them is their algorithmic design.

In the longitudinal CPM literature, the terms machine learning, data mining and statistical learning have all been used to refer to the following algorithms: regularised logistic regression (RLR), elastic net (EN), random forests, gradient boosting, support vector machines (SVM), artificial neural networks, and naïve Bayes (NB). Additional algorithms have been categorised under `matching’ algorithms for how they use repeated measurements for binary classification, where the conceptual interpretation is similar to that of the TC framework, please see Additional file 2 for further information*.* The majority of reported machine learning algorithms were employed to classify data for binary outcomes, with very limited attention on time-to-event outcomes [81–83].

Some of the algorithms stated above have been used in a TSM framework alongside other methods to capture the longitudinal covariate information, such as aggregation into summary statistics (RLR, EN, NB) [84–86], autoregressive time-series modelling (SVM) [87], Gaussian processes (SVM) [88] and temporal extraction [89]. The discussed methods are also often employed amongst an algorithm which performs variable selection, CPM development and performance assessment (internal validation) simultaneously [84].

The following subsections will provide a summary of the most common algorithms (temporal extraction, random forests, support vector machines, and artificial neural networks), and how they have been reported to incorporate longitudinal information in clinical risk prediction. All of the methods satisfy the third methodological aim (A3) and can account for the effect of covariate change on the event of interest.

##### Temporal extraction

Temporal extraction can be used to define different change types in repeated observation data such as ‘trends, statuses, and other complex time-related attributes’ [89]. The temporal patterns over time can correspond to 13 different temporal operators: BEFORE, OVERLAPS, MEETS, STARTS, DURING, and their inverse relations, as well as EQUALS [89]. A simpler version of this technique only consists of increasing, decreasing or stationary temporal processes [89]. Variations of this conceptual idea exist in the machine learning literature such as time interval related patterns [90, 91], and sequential pattern mining [83]. These algorithms are usually embedded into an algorithmic framework which aims to match patterns over time in a current patient to historical patient information and infer the probability of the outcome of interest [90, 92].

##### Random forests

Random forests are composed of a set of low-correlated decision trees developed upon subsets of data generated via bootstrap sampling [84, 93, 94]. A single decision tree can incorporate nonlinear relationships and interactions of classifications with a simple representation of the data [85]. Both random forests and gradient boosting are reported to be advantageous when a CPM requires a large number of predictors [84]. Gradient boosting is an extension of the random forests that iteratively generates a sequence of decision trees based on the misclassification of a previous decision tree [95]. Although random forests have been used with longitudinal data, it is unclear whether any dependence on time or the ordering of measurements has been acknowledged as it has recently been described as a time-independent method [6].

##### Support vector machines

Support vector machines (SVM) aim to maximise the distance between events and non-events in a high-dimensional space. SVMs explicitly divide the two domains with a linear or non-linear function, often estimated using a Kernel function [94, 96]. SVMs are less sensitive to outliers than standard logistic regression yet are more computationally intensive as they can harness high-dimensional covariate information [97].

While this approach has typically been combined (in a TSM framework) with aggregated data and time-series modelling techniques [87, 96, 97], SVMs have recently been employed as a one-stage approach for CPM development to harness repeated measurements in an observation window from EHR data [94]. It is thus implied that SVM can handle the longitudinal nature of predictor variables. However, time-dependency may have still been ignored as it has been reported that SVMs aggregate longitudinal information and ignore temporal relationships [98].

##### Artificial neural networks

Artificial neural networks (ANNs) are a complex mathematical model designed to replicate the decision making process of the human brain. However, unlike the tree-based algorithms described above, the network is designed inside a black box, also known as the hidden layers [85]. ANNs are specifically able to process nonlinear relationships amongst dependent and independent variables whose relationships are complex, multidimensional and interactive [85].

Artificial neural networks may be able to respect the structure of longitudinal data, yet this is unclear amongst the literature. Descriptions of the hierarchical extension of ANNs explicitly state that time-dependent covariates can be incorporated into the network, although no explicit applications are suggested [82, 99]. Recurrent neural networks (RNN) are extensions of ANNs that have the ability to remember historical results, establish relationships across repeated measurements and acknowledge patterns over time [98, 100]. Unlike articles discussing other ML techniques, articles using RNNs have been explicit about the method’s ability to harness high-dimensional data and tackle multivariate time-series problems for the prediction of a binary outcome [99, 101]. Clinically, RNNs were adopted to predict heart failure based on EHR data in 2018 [98], see Table 4.

## Discussion

This methodological review has identified three ways in which available methods can utilise longitudinal information to enhance the performance of CPMs: (A1) to better represent the predictor-outcome relationship; (A2) to infer a covariate value at a pre-specified time and (A3) to account for the effects of predictor change over time. All identified methods have been categorised into seven methodological frameworks which use longitudinal information in different ways: time-dependent covariate modelling; generalised estimating equations; landmark analysis; two-stage modelling; joint-modelling; trajectory classification and machine learning. Four of these frameworks can harness subject-level repeated measurements at the time of prediction for a new individual, as well as subject-level longitudinal information on a study population for CPM development.

Recent reviews of available methods for modelling repeatedly measured predictor variables in the development of CPMs have focussed on evaluating their predictive advantage over cross-sectional CPMs [5, 8–12, 14, 21, 102–106]. The range of compared methods varies across reviews, although joint models are typically compared with other methods. The choice of reviewed methods has often been determined by the specific methodological problem, such as modelling a single longitudinal predictor [5, 8, 12, 21], modelling multiple irregularly measured predictor variables prior to a fixed landmark time [9, 10], modelling multivariate longitudinal data in a critical care setting [11] or handling large datasets with small numbers of events [9]. Other reviews have been designed for comparing a newly proposed method with other available methods [21] or comparing more complex approaches with simpler methods [105, 106]. Finally, some reviews focus solely on the comparison of the LA and JM frameworks, which are the most popular approaches [12, 14, 102, 104].

Problematically, previous reviews and studies often refer to methods using different names. For example, TDCM (with a Cox proportional hazards model) has been reported as the ‘last observation carried forward’ approach for the way it handles repeated measurements at the time of prediction [5]. However, this is distinct from the ‘most recent observation’ approach discussed in another review, which refers to the application of a baseline CPM in EHR data [9]. Similarly, ‘ordinary regression calibration’ and ‘risk-set regression calibration’ methods have been defined as a sub-category of ME models as they have different assumptions for the random effects, yet these terms are not used elsewhere [5]. Therefore, we hope that this review will create a practical guide for researchers wishing to apply these methods, by providing a unified summary of the literature.

Welten et al. were the first reviewers to provide a set of available methods to address the methodological challenge and the practical implications of modelling repeated measurements for individual-level prediction [8]. However, the review focused on simple approaches (in the TSM framework), arguing that random-effects models are not appropriate for individual risk prediction in practice [8]. Nonetheless, it has now been argued that a new patient’s random effects can be estimated through Monte Carlo simulation [72, 73]. Plate et al. also proposed a framework to facilitate the understanding and uptake of a variety of multivariate longitudinal methods for prediction in critical care in 2019 [11]. Despite the authors advocating the framework to be more widely applicable to EHR-style data, the proposed framework was specified prior to the systematic database search, whereas the structure of this review’s output has been completely derived from the identified literature.

As the scope of the current methodological review was not restricted to a particular clinical application or a particular set of methods, a key strength of this study is its ability to provide a broader overview of available methodology, directly compare how methods use longitudinal information, and highlight some key considerations for applied researchers when choosing an appropriate method. These key considerations include, but are not restricted to, the type and amount of information available at the time of prediction (including the number and type of longitudinal predictors), how the CPM can benefit from the longitudinal information and what is known a priori to model development (i.e. imposed model assumptions).

During this review, considerations for future methodological research were also identified. The following aspects of CPM development were often overlooked within the current literature: sample size requirements, the handling of missing or irregularly-spaced data, effectively summarising longitudinal information, model validation (and avoiding statistical overfitting) and finally, how to quantify the improvement in predictive accuracy when incorporating repeated measurements. To elaborate, irregularly spaced measurements cannot be directly modelled using some methods, and so require additional imputation methods. Potential algorithms are emerging to choose the best way to summarise longitudinal trajectories in joint models [107], but there is limited discussion elsewhere around variable selection in a longitudinal context. Model validation techniques remain similar to those for cross-sectional CPMs where applicable, and the quantification of predictive improvement is often performed using differences in C-index which lacks clinical interpretability [108]. We recommend future research in each of these areas.

The limitations of our study should be kept in mind when interpreting its results. First, the systematic search employed to identify available methodology was designed using free-text. The evolving nature of this research space has resulted in a lack of uniformity in language when referring to repeated measurements of predictor variables and dynamic prediction amongst the literature. Second, the screening was performed by one author (LB) which may have introduced subjectivity and bias into the screening process. Third, while aggregate details of available software are provided in Table 4, the frequency and the level of detail of software reporting in the identified literature were not assessed.

The first limitation may have resulted in the systematic search missing some methods. To minimise this risk, the search in Table 1 was designed to cover all methodological purposes of longitudinal data for clinical risk prediction, including prediction of population-level change, the identification of predictors and methods to address calibration drift. Furthermore, an initial title screening with a much broader search strategy was performed to identify further relevant articles from which key words and terminology could be extracted. To reduce the risk of subjectivity as a result of the second limitation, the reviewer remained cautious about articles where it was unclear whether they fit the inclusion criteria and carried them forward to the next round of screening. Despite a general lack of detail in the literature in the reporting of available software for discussed methods, all reported available software has been included in Table 4. Such information will be useful for the implementation of identified methods and can provide an indication of where software may not have been well-reported.

Having compared how methods use longitudinal information, summarised their reported advantages and disadvantages and grouped them based on methodological approach, we hope to facilitate the understanding of a broad and complex research domain. The findings from this review consolidate the message from previous reviews, that there is no straight-forward approach to developing a longitudinal CPM. However, to reiterate, the choice of methods is substantially reduced by the following considerations: the type and amount of information available at the time of prediction (including the number and type of longitudinal predictors), how the CPM can benefit from the longitudinal information and the validity of any assumptions for a specific application.

## Conclusions

We have grouped methods available for incorporating repeatedly measured predictor variables into the development of a CPM, identified their methodological aims and discussed their reported advantages and limitations. In addition, amongst the literature we found some key considerations for CPM development and identified opportunities for further methodological research. Most importantly, however, our review has identified seven methodological frameworks which offer a wide range of ways in which longitudinal information can enhance CPMs by improving the representation of a predictor-outcome relationship, updating predictions during follow-up, inferring covariate values, or accounting for the effect of how a predictor variable changes over time.

## Supplementary information

**Additional file 1:** Table 1 Reference List. Title of data: Journal articles included in the review, ordered alphabetically by first author surname. Description of data: A list of all articles included in the review, including author names, title of publication, journal of publication, volume, pages and DOI number.

**Additional file 2: Additional Results.** Title of data: Additional methods identified in the review. Description of data: Further details about methodology discussed within the review.

## Data Availability

Data sharing not applicable to this article as no datasets were generated or analysed during the current study.

## Abbreviations

CPM: Clinical prediction model
CVD: Cardiovascular disease
EHR: Electronic health record
TDCM: Time-dependent covariate modelling
GEE: Generalised estimating equations
LA: Landmark analysis
TSM: Two-stage modelling
JM: Joint modelling
TC: Trajectory classification
ML: Machine learning
LOCF: Last observation carried forward
ME: Mixed effect
LMM: Linear mixed model
SRE: Shared random effect
JLCM: Joint latent class model
JFM: Joint frailty model
RLR: Regularised logistic regression
EN: Elastic net
SVM: Support vector machine
ANN: Artificial neural network
RNN: Recurrent neural network
MCMC: Markov chain Monte Carlo
