# Supplementary material for: Harnessing repeated measurements of predictor variables for clinical risk prediction: a review of existing methods
Source: Diagn Progn Res. 2020 Jul 9;4:9. doi: 10.1186/s41512-020-00078-z (PMC7346415; doi:10.1186/s41512-020-00078-z)
Supplement: Supplementary file 2 — Additional file 2: Additional Results. Title of data: Additional methods identified in the review. Description of data: Further details about methodology discussed within the review. [file 41512_2020_78_MOESM2_ESM.pdf]

Additional File 1 – Additional Results

Appendix 1: Functional principal components and `time series' applied in two-stage modelling framework

Functional principal components

Functional principal components (FPC) analysis is an extension of principal components analysis for temporal or spatial longitudinal information (1, 2). The method assumes a true subject-specific latent (unobserved) trajectory for each predictor in the CPM (1). The use of FPC analysis for longitudinal CPMs is similar to that of ME modelling in that the rate of change, or covariate process is captured in the subject-specific random effects. More specifically in FPC analysis, the method defines different characteristics of the covariate trajectory, for each individual, in a set of K FPC scores (1, 3). The number of components K can be determined by the proportion of variance explained by the first K components (i.e. the first K components which explain 95% of the variance) (3). These K FPC scores can then be entered into a binary or time-to-event model (TS framework) (1, 3). The primary aim of the method is to capture variations and patterns in the covariate trajectory within and between individuals in the cohort (1, 2).

FPC analysis produces a non-parametric function to summarise individual covariate trajectories over time. This non-parametric function is made up of a population-level mean trajectory, K eigen functions which reflect pattern variations, and their corresponding K FPC score for each individual (1-4). As individual deviations from the population mean trajectory are captured in the FPC scores, these are the only aspects which are then carried over into a CPM for event prediction (1, 3). Using parameters which have already been estimated on the model development cohort, the FPC scores for a new individual can be calculated in the same way as it is for model development (1).

The key difference between ME modelling and FPC analysis is that no assumption is required about the shape or trend of the covariate trajectory, this avoids any loss of predictive accuracy from miss-specified parametric functions (1, 2). FPC analysis is particularly advantageous when longitudinal measurements are sparse and irregularly measured (1-3).

One of the limitations is that the FPC scores and covariate trends cannot be graphically presented on the same scale as the true values, but the eigen functions presented are linearly proportional to the true values (1). This means that the covariate pattern would be identical to that of the eigen function derived from the FPC analysis.

The FPC analysis approach has recently been extended to consider multivariate longitudinal trajectories which may be highly-correlated with each other (3). It has also been extended to the TDCM framework with time-varying coefficients, to allow for updated predictions using moving time windows (2, 4, 5).

Time series

Three-level hierarchical autoregressive models have been proposed to harness irregularly-measured covariate values for individual patients, to infer the covariate values at regularly-spaced time points, and to account for random interpatient and residual variability in the data (6). These were used in conjunction with a Cox regression model and an Accelerated failure-time (AFT) model (6).

The Kalman Filter, has been employed in CPM development to remove uninformative noise and measurement error from the underlying trajectory, and to infer the true value of a covariate at the time of prediction (7). The Kalman filter is able to identify multi-dimensional latent states through which the underlying process transitions, and uses previous measurements to infer the true latent state of the predictor variable at prediction time.

The Kalman Filter offers more flexibility in the specification of the underlying trajectory than a linear mixed model, and therefore could be a preferred method for longitudinal covariates with a highly-variable continuous outcome.

Appendix 2: Joint modelling variations

This section will describe and differentiate between the three different variations of joint models used for clinical risk prediction.

The shared random effects joint model

The first and most popular type of joint model found in the literature for incorporating longitudinal information into clinical risk prediction is called the shared random effects (SRE) model (8). The traditional SRE model would only include the random effects themselves within the survival or binary event sub-model (9-11); however these can become difficult to interpret when enhanced mixed-effects models are used to model the covariate trajectory (12).

The distinct features of the SRE framework are that it assumes conditional independence between the longitudinal and survival outcomes on the random-effects, it assumes a homogenous population, and the random-effects from the mixed-effects model specification capture both the correlation between within-subject measurements as well as the dependency between the longitudinal covariate and the outcome of interest (8, 14).

Extensions of this approach involve including predicted values of the longitudinal covariate in a time-dependent survival model, as well as other association structures such as the trajectory slope at a pre-specified time (15-17), or the area under the trajectory (18-20). These model extensions are required to be chosen a priori because the computational intensity of joint modelling specification limits the use of automatic variable selection algorithms (21). Rizopoulos (2014) proposed a Bayesian Model Average algorithm to allow predictions to be made for an individual from multiple SRE models with the different association structures described above (19, 22).

Albert (2012) also proposed a computationally attractive two-stage SRE framework to predict a binary event from repeated measurements of a single covariate by using a *probit* model to link the longitudinal and binary outcome, and a simple Gaussian assumption for the random effects (9). However, if the random effects could derive from a mixture of normal distributions then numerical optimisation techniques are required such as Newton-Raphson algorithm, or multivariate Gaussian quadrature (9). A more generalised approach to this framework for multivariate longitudinal outcomes has also been introduced by Kim et al. in 2012 (11).

The SRE JM has also been further extended to model time-dependent random-effects, time-varying coefficients (18), and include a third sub-model alongside the longitudinal and survival models to acknowledge non-ignorable missing data or an underlying cure fraction (23-26).

SRE model specification can be performed under both a frequentist (Newton-Raphson or Expectation-Maximisation algorithm) or under a Bayesian framework (using the Markov Chain Monte Carlo algorithm). Estimation processes for the SRE model are distinct to the below Joint Latent Class Model. Shared Random Effects (SRE) joint models can be implemented using the JM and JMbayes packages on R.

<u>The joint latent class model</u>

Proust-Lima and Taylor proposed a *Joint latent class model* (JLCM) for CPM development in 2009, the proposed model is reported to be more computationally-efficient than the SRE model (8, 14). Unlike the shared random effects (SRE) model which assumes conditional independence between the covariate trajectory and the time-to-event outcome on the random-effects, this method conditions independence on a latent class structure (8, 14).

The survival probability over the next *t* years is solely dependent on the probabilities of belonging to each latent class rather than the specific covariate trajectory characteristics (14). The probability that an individual belongs to each latent class is conditioned on subject-specific covariates, and is usually modelled using a multinomial logistic regression model. The longitudinal covariate information is modelled using a mixed-effect model just like the SRE joint models described above. However, for this framework, the random effects for each individual are also dependent on the latent class group.

Unlike the SRE model, the random effects in the ME model only capture the within-subject correlation between measurements, and the latent classes capture the dependency between the longitudinal covariate and the outcome of interest (8). Moreover, this framework assumes a heterogeneous population rather than a homogeneous one, which could be divided into a collection of latent classes (8). This approach has been extended to incorporate multivariate longitudinal information (27, 28), and with multi-state models to inform screening strategies (29).

Although this framework appears to have had little attention in comparison to the SRE model for CPM development, it is reported to hold some advantages over the SRE specifically for clinical risk

prediction (8). The main reported advantage of this framework is that it is computationally more efficient as the integration over the random effects which is required for the SRE model, is replaced by a summation over the latent classes (8). However, it is also reported that the estimation process would need to be repeated to ensure convergence and to choose an appropriate number of latent classes (8). Another reported advantage is that the JLCM framework is designed to describe the observations without specific assumptions about the association a priori (8). One of the main drawbacks of the SRE model for individual risk prediction is that the random effects specification needs to be correctly specified (9).

Specifically for the JLCM, the model parameters can be estimated using maximum likelihood estimation from the Marquardt algorithm but strict convergence criteria are required, more details can be found here (8). Bayesian estimation processes are limited here as the latent-class specific parameters need to be permutated, this does not cause any problems for maximum likelihood estimation however (8). Joint Latent Class Models (JLCMs) can be implemented using the *lcmm* package on R.

<u>The joint frailty model for recurrent events</u>

Joint frailty models (JFM) are distinct to the joint models described above because their focus is on simultaneously modelling recurrent events and a terminal event. A traditional JFM will not include a sub-model for the covariate trajectory, but a model for recurrent events, so a proportional hazards model on each interval between events, as well as a proportional hazards model for the terminal event (30, 31). These two models will share what is referred to as a "frailty" term which is a random effect that can account for the correlation between recurrent events, and/or the association between the two different outcome types (31).

Although JFMs as a stand-alone framework are not incorporating longitudinal information in the same way as the two frameworks described above, conceptually they are modelling an informative process and how this is associated with the outcome of interest. Extensions to incorporate covariate trajectory modelling as well as recurrent events to predict the risk of a terminal event have been proposed (31). This involves a third sub-model for the covariate trajectory which could be specified in the same way as before, including nonlinear mixed-effects models (32).

Various versions of the Gaussian-Hermite quadrature algorithm are available for model specification depending on required computational efficiency (31). All applications discovered by the systematic search had used the *frailtypack* package on R (30-35). The *frailtyPenal* function in this package allows for frailty models to be used for interval-censored and clustered outcomes whilst also accounted for left-truncation caused by delated entry (35).

Appendix 3: Machine learning algorithms – Additional information

Gradient/Adaptive Boosting

Gradient Boosting iteratively generates a sequence of decision trees, based on the misclassification of individuals by a previous decision tree (36). More specifically, the algorithm will design the first decision tree for a known outcome of interest, and then the next decision tree will be designed to identify individuals who were not correctly identified to experience the outcome of interest by the first tree (36) . Each additional tree aims to better identify high-risk individuals, the process continues iteratively (36). K-fold cross-validation can be used to determine the optimal number of trees (36, 37). (Available software: *Adaboost* or *gbm* R packages).

Multiple Measurements Support Vector Machine (MMSVM)

The support vector machine has been used in conjunction with a data merging algorithm which aims make use of multiple measurements of a single predictor by prioritising a single measurement of a predictor in a specific time-window and maintaining summary statistics of all measurements taken in the observation window to prevent loss of information (38). Although this approach harnesses repeated measurements until the time of the outcome of interest to enhance classification, it is unclear how beneficial this approach would be for predicting future risk for new individuals who do not have any future longitudinal information.

Support Vector Machine on Time-Series Model (SVMTSM)

SVMs can be used in conjunction with autoregressive modelling techniques to capture time dependency and within-subject correlation (39). These two methods have worked together in Alzheimer's disease, where the autoregressive model was used to predict future values of a covariate and then the SVM was applied to classify the trajectories for events and non-event subjects (39).

Multiple Kernel Learning and Random-effects

The standard SVM techniques have been further extended to incorporate correlations between different data sources and amongst repeated within-subject measurements (40). Subject-specific short-term and long-term random effects are modelled using kernel functions to acknowledge within-subject correlation and variability (40). This technique has been reported to conceptually be similar to semiparametric or nonparametric mixed-effects models depending on the choice of kernel, however the proposed method is said to provide greater flexibility through kernels and can easily be scaled up to high-dimensional data (40). The theory behind this extension of SVM has been described elsewhere (40).

Hierarchical Neural Networks

Walker and Musen, (41), introduced a hierarchical structure for an ANN model to exploit censored and time-varying information. The illustrations in this paper suggest that time-dependent covariates can be handled by the ANN framework (41). However, in addition to this application, the

hierarchical ANN can be employed on a conditional survival framework where you construct a separate ANN at pre-defined time points for the remaining at-risk population and take into account the most recent subject-specific covariate information (42).

Sequential Neural Networks

A sequential multi-layered perception (SMLP) neural network with backpropagation learning, just like the RNN models, aims to harness the time-sensitivity of covariate information, but also the timing of event occurrence (43). It was recently applied to capture the time-dependent nature of risk factors as predictors to the occurrence of diabetes (43). The details of how this algorithm works are explained elsewhere (43), but it has been reported to be similar to that of the TDCM approach in survival analysis yet the data used for modelling are the incremental changes rather than the covariate values. The structure of the SMLP learns from a small change in the input and produces a prediction according the change (43). Therefore, risk predictions can be updated over time whilst also accounting for predictions made by the algorithm at a previous time step.

Unlike the RNN model, this method has been proposed specifically for a clinical application. On the other hand, a possible limitation of the proposed algorithm for EHR-style data is that it excludes any variables which have over 50% missing data (43).

3.3.7.5 Matching algorithms

Matching algorithms involve measuring the similarity of subject-level trajectories to historical trajectories from model development data. Conceptually, in how these approaches use longitudinal information, they map onto the TC framework. These algorithms include: a combination of mathematical modelling and an expert advice algorithm (36), multiple measurements case-based reasoning (37), and an interval mining algorithm (44, 45). More specifically, as an example, the multiple measurement case-based reasoning approach conceptually matches a new patient to an existing patient based on the frequency, timings and values of measurements (36, 37).

References

1. Fang HB, Wu TT, Rapoport AP, Tan M. Survival analysis with functional covariates for partial follow-up studies. Statistical Methods in Medical Research. 2016;25:2405-19.

2. Lin X, Li R, Yan F, Lu T, Huang X. Quantile residual lifetime regression with functional principal component analysis of longitudinal data for dynamic prediction. Stat Methods Med Res. 2018:962280217753466.

3. Li K, O'Brien R, Lutz M, Luo S, Alzheimers Dis Neuroimaging I. A prognostic model of Alzheimer's disease relying on multiple longitudinal measures and time-to-event data. Alzheimers & Dementia. 2018;14:644-51.

4. Yan F, Lin X, Li R, Huang X. Functional principal components analysis on moving time windows of longitudinal data: dynamic prediction of times to event. Journal of the Royal Statistical Society Series C: Applied Statistics. 2018;67:961-78.

5. Yan FR, Lin X, Huang XL. DYNAMIC PREDICTION OF DISEASE PROGRESSION FOR LEUKEMIA PATIENTS BY FUNCTIONAL PRINCIPAL COMPONENT ANALYSIS OF LONGITUDINAL EXPRESSION LEVELS OF AN ONCOGENE. Annals of Applied Statistics. 2017;11:1649-70.

6. Chi CL, Zeng W, Oh W, Borson S, Lenskaia T, Shen X, et al. Personalized long-term prediction of cognitive function: Using sequential assessments to improve model performance. Journal of Biomedical Informatics. 2017;76:78-86.

7. Perotte A, Ranganath R, Hirsch JS, Blei D, Elhadad N. Risk prediction for chronic kidney disease progression using heterogeneous electronic health record data and time series analysis. Journal of the American Medical Informatics Association. 2015;22:872-80.

8. Proust-Lima C, Sene M, Taylor JMG, Jacqmin-Gadda H. Joint latent class models for longitudinal and time-to-event data: a review. Statistical Methods in Medical Research. 2014;23:74-90.

9. Albert PS. A linear mixed model for predicting a binary event from longitudinal data under random effects misspecification. Statistics in Medicine. 2012;31:143-54.

10. Qiu F, Stein CM, Elston RC, TBRU TBRU. Joint modeling of longitudinal data and discrete-time survival outcome. Statistical Methods in Medical Research. 2016;25:1512-26.

11. Kim S, Albert PS. A Class of Joint Models for Multivariate Longitudinal Measurements and a Binary Event. Biometrics. 2016;72:917-25.

12. Li K, Furr-Stimming E, Paulsen JS, Luo S. Dynamic prediction of motor diagnosis in Huntington's disease using a joint modeling approach. Journal of Huntington's Disease. 2017;6:127-37.

13. Liu DP, Albert PS. Combination of longitudinal biomarkers in predicting binary events. Biostatistics. 2014;15:706-18.

14. Proust-Lima C, Taylor JMG. Development and validation of a dynamic prognostic tool for prostate cancer recurrence using repeated measures of posttreatment PSA: a joint modeling approach. Biostatistics. 2009;10:535-49.

15. Taylor JM, Park Y, Ankerst DP, Proust-Lima C, Williams S, Kestin L, et al. Real-time individual predictions of prostate cancer recurrence using joint models. Biometrics. 2013;69:206-13.

16. Yang L, Yu M, Gao S. Prediction of coronary artery disease risk based on multiple longitudinal biomarkers. Statistics in Medicine. 2016;35:1299-314.

17. Rizopoulos D. Dynamic Predictions and Prospective Accuracy in Joint Models for Longitudinal and Time-to-Event Data. Biometrics. 2011;67:819-29.

18. Andrinopoulou ER, Eilers PHC, Takkenberg JJM, Rizopoulos D. Improved dynamic predictions from joint models of longitudinal and survival data with time-varying effects using P-splines. Biometrics. 2018;74:685-93.
19. Andrinopoulou ER, Rizopoulos D, Takkenberg JJM, Lesaffre E. Combined dynamic predictions using joint models of two longitudinal outcomes and competing risk data. Statistical Methods in Medical Research. 2017;26:1787-801.
20. Sweeting MJ. Using predictions from a joint model for longitudinal and survival data to inform the optimal time of intervention in an abdominal aortic aneurysm screening programme. Biometrical Journal. 2017;59:1247-60.
21. Pauler DK, Finkelstein DM. Predicting time to prostate cancer recurrence based on joint models for non-linear longitudinal biomarkers and event time outcomes. Statistics in Medicine. 2002;21:3897-911.
22. Rizopoulos D, Hatfield LA, Carlin BP, Takkenberg JJM. Combining Dynamic Predictions From Joint Models for Longitudinal and Time-to-Event Data Using Bayesian Model Averaging. Journal of the American Statistical Association. 2014;109:1385-97.
23. Yu MG, Taylor JMG, Sandler HM. Individual prediction in prostate cancer studies using a joint longitudinal survival-cure model. Journal of the American Statistical Association. 2008;103:178-87.
24. Piulachs X, Alemany R, Guillen M, Rizopoulos D. Joint models for longitudinal counts and left-truncated time-to-event data with applications to health insurance. Sort-Statistics and Operations Research Transactions. 2017;41:347-71.
25. Zhang N, Chen H, Zou Y. A joint model of binary and longitudinal data with non-ignorable missingness, with application to marital stress and late-life major depression in women. Journal of Applied Statistics. 2014;41:1028-39.
26. Zhang S, Mueller P, Do K-A. A Bayesian Semiparametric Survival Model with Longitudinal Markers. Biometrics. 2010;66:435-43.
27. Proust-Lima C, Letenneur L, Jacqmin-Gadda H. A nonlinear latent clss model for joint analysis of multivariate longitudinal data and a binary outcome. Statistics in Medicine. 2007;26:2229-45.
28. Proust-Lima C, Dartigues JF, Jacqmin-Gadda H. Joint modeling of repeated multivariate cognitive measures and competing risks of dementia and death: A latent process and latent class approach. Statistics in Medicine. 2016;35:382-98.
29. Li H, Gatsonis C. Dynamic optimal strategy for monitoring disease recurrence. Science China-Mathematics. 2012;55:1565-82.
30. Katki HA, Cheung LC, Fetterman B, Castle PE, Sundaram R. A joint model of persistent human papilloma virus infection and cervical cancer risk: implications for cervical cancer screening. Journal of the Royal Statistical Society Series a-Statistics in Society. 2015;178:903-23.
31. Krol A, Ferrer L, Pignon JP, Proust-Lima C, Ducreux M, Bouche O, et al. Joint model for left-censored longitudinal data, recurrent events and terminal event: Predictive abilities of tumor burden for cancer evolution with application to the FFCD 2000-05 trial. Biometrics. 2016;72:907-16.
32. Krol A, Tournigand C, Michiels S, Rondeau V. Multivariate joint frailty model for the analysis of nonlinear tumor kinetics and dynamic predictions of death. Statistics in Medicine. 2018;37:2148-61.

33. Lafourcade A, His M, Baglietto L, Boutron-Ruault MC, Dossus L, Rondeau V. Factors associated with breast cancer recurrences or mortality and dynamic prediction of death using history of cancer recurrences: The French E3N cohort. BMC Cancer. 2018;18 (1) (no.
34. Mauguen A, Rachet B, Mathoulin-Pelissier S, MacGrogan G, Laurent A, Rondeau V. Dynamic prediction of risk of death using history of cancer recurrences in joint frailty models. Statistics in medicine. 2013;32:5366-80.
35. Rondeau V, Mauguen A, Laurent A, Berr C, Helmer C. Dynamic prediction models for clustered and interval-censored outcomes: Investigating the intra-couple correlation in the risk of dementia. Statistical Methods in Medical Research. 2017;26:2168-83.
36. Koyner JL, Carey KA, Edelson DP, Churpek MM. The Development of a Machine Learning Inpatient Acute Kidney Injury Prediction Model. Critical Care Medicine. 2018;46:1070-7.
37. Konerman MA, Zhang Y, Zhu J, Higgins PDR, Lok ASF, Waljee AK. Improvement of predictive models of risk of disease progression in chronic hepatitis C by incorporating longitudinal data. Hepatology. 2015;61:1832-41.
38. Tseng YJ, Ping XO, Liang JD, Yang PM, Huang GT, Lai FP. Multiple-Time-Series Clinical Data Processing for Classification With Merging Algorithm and Statistical Measures. Ieee Journal of Biomedical and Health Informatics. 2015;19:1036-43.
39. Minhas S, Khanum A, Riaz F, Khan SA, Alvi A, Alzheimers Dis Neuroimaging I. Predicting Progression From Mild Cognitive Impairment to Alzheimer's Disease Using Autoregressive Modelling of Longitudinal and Multimodal Biomarkers. IEEE Journal of Biomedical and Health Informatics. 2018;22:818-25.
40. Chen T, Zeng D, Wang Y. Multiple kernel learning with random effects for predicting longitudinal outcomes and data integration. Biometrics. 2015;71:918-28.
41. Ohno-Machado L, Walker MG, Musen MA. Hierarchical neural networks for survival analysis. Medinfo. 1995;8 Pt 1:828-32.
42. Parmanto B, Doyle HR. Recurrent neural networks for predicting outcomes after liver transplantation: representing temporal sequence of clinical observations. Methods of Information in Medicine. 2001;40:386-91.
43. Park J, Edington DW. A sequential neural network model for diabetes prediction. Artificial Intelligence in Medicine. 2001;23:277-93.
44. Goyal D, Tjandra D, Migrino RQ, Giordani B, Syed Z, Wiens J. Characterizing heterogeneity in the progression of Alzheimer's disease using longitudinal clinical and neuroimaging biomarkers. Alzheimer's & Dementia : Diagnosis, Assessment & Disease Monitoring. 2018;10:629-37.
45. Ajemba PO, Ramirez L, Durdle NG, Hill DL, Raso VJ. A support vectors classifier approach to predicting the risk of progression of adolescent idiopathic scoliosis. Ieee Transactions on Information Technology in Biomedicine. 2005;9:276-82.
